# Books Reviewed

**Published:** 1867-07

**Authors:** 


### Books Reviewed.

Watson Abridged. By J. J. Meyler, A. M., M. D.

We believe that in no way can we convey a better idea of the character of this book to our readers than by making the following quotations from the preface:— "The merits of this Abridgment and the advantages claimed for it are: 1, that it is of pocket size; 2, that it contains everything of importance to be found in the large work; 3, that the lectures, being short, can be read in a few minutes; 4, that the matter of each lecture is divided, according to the subject into parts, by such side heads as Symptoms, Treatment, Causes, Diagnosis, Prognosis, etc., thus rendering it easy to obtain, at a glance, any required information; 5, that being numbered as in the large work, the lectures can readily be compared with the original; 6, that in addition to the various tables, and the List of Poisons, their Symptoms and Treatment, it contains a short account of the Uses, Preparation, Doses (taken from the U. S. Dispensatory) of the many medicines mentioned in this work." We have carefully examined many of the "Abridged Lectures," being greatly pleased with the clear and connected ideas, obtained from their perusal, and to the use of students this work is especially adapted as a convenient and expeditious means of reference during the lecture seasons.

Guide for using Medical Batteries, (Electricity and Nervous Diseases,) showing the most improved Apparatus, Methods and Rules for the Medical Employment of Electricity in the Treatment of Nervous Diseases. By Alfred C. Garrett, M. D.

Not until quite recently has the attention of the medical profession been directed to consider electricity as a remedial agent, and it was with a view of pointing out the important relations which electricity bears to most diseases of the nervous system, as also to many complicated, chronic and obscure affections, that the author introduced his work on "Medical Electricity and Nervous Diseases," giving in it practical directions as to *where*, *when*, and *how* to employ electricity as a remedy. The present volume, a compendium from his larger work, is intended to serve as a concise and practical guide to the medical and surgical uses of electrical apparatus, giving the most simple and effective methods and rules for their use and safety. The extensive experience of Dr. Garrett as a teacher, and the many years of long and patient reseaches in this branch of medical science have thoroughly acquainted him with the wants in this field of our literature, and although many of the subjects embodied may be found in ordinary works on natural philosophy, much pleasure and profit will be derived by the student from the study of this work.

Report to the International Sanitary Conference of a commission from that body on the Origin, Endemnicity, Transmissibility and Propagation of Asiatic Cholera. Translated by Samuel L. Abbott, M. D., Physician to the Massachusetts General Hospital, etc.

We have had opportunity on a former occasion (Vol. vi, No. 1,) to place before our readers a brief synopsis of this report, thus rendering an extended notice on our part, which the importance of the questions therein involved, would otherwise require, unnecessary. We are unacquainted with any work in our literature which treats the origin, endemnicity, transmissibility and propagation of Asiatic cholera, more comprehensively and more unbiased by personal opinions than the report of the members of this commission, and the opinions entertained therein, we have no doubt will exert a powerful influence upon the medical profession throughout the civilized world. To the translator, Dr. Samuel L. Abbott, the thanks of American physicians are due for the early publication, and masterly translation of this important document.

---

A Theory of Inflammation—Its Cause, Course and Rationale of Treatment. By Nelson L. North, M. D.

The views advanced in this monograph, considering the pathological changes of inflammation as dependent on a perverted action of nervous influence upon the process of nutrition, were expressed by the author in his inaugural thesis, and which views being strengthened into a conviction by thirteen years of careful observation induces him to give publicity to them. The recent progress in physiological and pathological truths, strongly tend to the confirmation of this theory, and although we do not believe in speculations unsupported by experimental observations, we do consider time well employed in the perusal of this pamphlet.

---

Accidental and Congenital Atresia of the Vagina, with a mode of Operating for Successfully Establishing the Canal.

The usual mode of dividing the adhesions by the knife has proved so unsatisfactory in its result, owing to the rapid granulations of incised wounds, that the author has determined upon a new mode of procedure. The cicatrical line is carefully divided by scissors, and the adhesions either gradually broken down by the finger or divided by the scissors according to circumstances, the opening of the vagina being maintained by a glass plug of various dimensions, and open at the outer end. The author refers to two cases of atresia, in one of which, the operation for establishing the vaginal canal, had proved unsuccessful, after five attempts operated upon in usual manner, the result of which was eminently satisfactory when treated by the afore-described method.

---

Journal of Psychological Medicine and Medical Jurisprudence.

The first number of the abovementioned quarterly journal, under the able supervision of Dr. W. A. Hammond, has been placed upon our table. It contains original articles on the Physiology and Pathology of the Mind and Nervous System, and Medical Jurisprudence, Selections and Reviews. The want of a medical journal upon these subjects has been keenly felt by the profession, and its editor who, for many years has successfully labored in the study of the diseases of the mind, the result of which labors being two volumes of great merit, besides many pamphlets, is eminently qualified to conduct this enterprise most successfully.— The publishers, Messrs. A. Simpson & Co., have presented the Journal in the most attractive style. We welcome our new exchange, wishing it such success as will place its existence upon a permanent basis.

